# Co-occurring Fatigue and Lymphatic Pain Incrementally Aggravate Their Negative Effects on Activities of Daily Living, Emotional Distress, and Overall Health of Breast Cancer Patients

**DOI:** 10.1177/15347354221089605

**Published:** 2022-04-21

**Authors:** Mei Rosemary Fu, Melissa L. McTernan, Jeanna M. Qiu, Christine Miaskowski, Yvette P. Conley, Eunjung Ko, Deborah Axelrod, Amber Guth, Tamara J. Somers, Lisa J. Wood, Yao Wang

**Affiliations:** 1 Rutgers University, Camden, NJ, USA; 2 Boston College, Chestnut Hill, MA, USA; 3 Harvard Medical School, Boston, MA, USA; 4 University of California, San Francisco, CA, USA; 5 University of Pittsburgh, Pittsburgh, PA, USA; 6 The Ohio State University, Columbus, OH, USA; 7 New York University School of Medicine, New York, NY, USA; 8 Duke University School of Medicine, Durham, NC, USA; 9 New York University Tandon School of Engineering, Brooklyn, NY, USA

**Keywords:** activities of daily living, breast cancer, emotional distress, fatigue, health, lymphatic, pain

## Abstract

**Background:**

Fatigue and lymphatic pain are the most common and debilitating long-term adverse effects of breast cancer treatment. Fatigue and pain independently have negative effects on quality of life, physical functions, and cancer recurrence-free survival. The interactions between fatigue and pain may aggravate their negative effects.

**Objectives:**

Examine the effects of co-occurring fatigue and lymphatic pain on activities of daily living (ADLs), emotional distress, and overall health of breast cancer patients.

**Methods:**

A cross-sectional and observational design was used to enroll 354 breast cancer patients. Valid and reliable instruments were used to assess fatigue, lymphatic pain, ADLs, emotional distress, and overall health. Descriptive statistics and multivariable regression models were used for data analysis.

**Results:**

After controlling for demographic and clinical factors, patients with co-occurring fatigue and lymphatic pain had higher odds of having impaired ADLs (OR = 24.43, CI = [5.44-109.67], *P* < .001) and emotional distress (OR = 26.52, CI = [9.64-72.90], *P* < .001) compared to patients with only fatigue and only lymphatic pain. Patients with co-occurring fatigue and lymphatic pain had 179% increase in impaired ADL scores (*B* = 8.06, CI = [5.54-10.59]) and 211% increase in emotional distress scores (*B* = 9.17, CI = [5.52-12.83]) compared to those without co-occurring fatigue and lymphatic pain. Patients with co-occurring fatigue and lymphatic pain had a 34% decrease (*B* = −26.29, CI = [−31.90 to −20.69]) and patients with only fatigue had a 33% decrease in overall health scores (*B* = −25.74, 95% CI = [−34.14 to −17.33]), indicating poor overall health.

**Conclusions:**

Fatigue and lymphatic pain affected 66.4% of breast cancer patients. Findings from this study suggest that co-occurring fatigue and lymphatic pain have negative effects on breast cancer patients’ ADLs, emotional distress, and overall health. The synergistic interactions between fatigue and lymphatic pain incrementally aggravated their negative effects on ADLs and emotional distress. Findings of the study highlight the need to evaluate the underlying mechanisms for co-occurring fatigue and lymphatic pain and develop interventions that target both fatigue and lymphatic pain to improve breast cancer patients’ the quality of life.

## Introduction

With advances in diagnosis and treatments for breast cancer, the 5-year survival rate for these patients has increased up to 90%.^1-2^ However, many breast cancer patients experience long-term adverse effects of cancer treatment. Fatigue and lymphatic pain are the most common and debilitating long-term adverse effects that negatively impact patients’ quality of life (QOL) as well as cancer recurrence-free survival.^2-6^ Compared to other types of cancer, patients treated for breast cancer have more lost disability-adjusted life years.^2^ Persistent fatigue and lymphatic pain may be 2 of the adverse effects that contribute to lost disability-adjusted life years in these patients.^3,6^

Cancer-related fatigue is defined as a sense of physical, emotional, and/or cognitive tiredness or exhaustion that is not proportional to recent activity and interferes with usual functioning due to cancer or cancer treatment.^7,8^ While fatigue usually subsides after the completion of treatment, more than 40% of patients experience persistent fatigue even years after the completion of cancer treatment.^9^ Lymphatic pain is defined as a variety of pain sensations (ie, pain, aching, soreness) in the ipsilateral upper limb or body due to an accumulation of lymph fluid from a compromised lymphatic system after cancer treatment.^4-6^ Lymphatic pain occurs most common in patients with a diagnosis of lymphedema,^7^ however, more than 50% of patients without a diagnosis of lymphedema also report lymphatic pain.^6,10,11^ Lymphedema is defined as an increased limb size or girth in the ipsilateral upper limb.^5,10,11^ For patients without a diagnosis of lymphedema, the experience of lymphatic pain indicates an early stage of lymphedema because lymphatic pain often precedes changes in limb size or girth and a lymphedema diagnosis.^6,11^ Patients who report pain on the affected ipsilateral upper limb or body are nearly twice as likely to develop lymphedema.^11^ Risk factors for fatigue and lymphatic pain are similar, including demographic characteristics (eg, age, body mass index [BMI], ethnicity, marital status, level of education, and employment status) and clinical characteristics (eg, type of cancer surgery, type of lymph node procedure, number of lymph nodes removed, receipt of radiation and/or chemotherapy, and years since breast cancer diagnosis). In addition, fatigue and lymphatic pain are inflammatory conditions^5,7,9^ and both are the most common and debilitating long-term adverse effects after cancer treatment.^2-6^ It is important to investigate if these 2 adverse symptoms occur concurrently.

While breast cancer patients report the co-occurrence of fatigue and pain, most studies have investigated each symptom separately. Across these studies, fatigue^12,13,14^ and pain^15^ had negative effects on physical activity, QOL, and survival. However, no studies have examined the effects of co-occurring fatigue and lymphatic pain on activities of daily living (ADLs), emotional distress, and overall health of breast cancer patients. The interaction between fatigue and pain may aggravate poor health conditions, negative emotions, decreased physical function, and even multi-organ toxicity.^16^ Therefore, the purpose of this study was to investigate the effects of co-occurring fatigue and lymphatic pain on ADLs, emotional distress, and overall health of breast cancer patients. We hypothesized that co-occurring fatigue and lymphatic pain would have incremental negative effects on patients’ ADLs, emotional distress, and overall health.

## Methods

### Ethical Consideration

This analysis is part of a larger study (IRB s16-01665) approved by the Institutional Review Board of New York University (NYU) Langone Health, in New York City of the United States. The protection of human subjects was ensured by following the guidelines set forth by the Institutional Review Board. Written informed consent was obtained from each patient.

### Study Design

A cross-sectional and observational design was used.

### Setting

This study was conducted in a nursing research laboratory located in the breast cancer clinic of NYU Perlmutter Cancer Center, a National Cancer Institute designated Cancer Center in New York City, United States.

### Study Participants

The sample consisted of female patients (n = 354) who were older than 21 years of age; had completed acute treatment (ie, surgery, radiation, chemotherapy) for breast cancer greater than 3 months before enrollment; and had no signs of metastatic disease or recurrence. Women were excluded if they had: (a) renal or heart failure, cardiac pacemaker or defibrillator, artificial limbs, or were pregnant because accurate measurement of body mass index (BMI) may not be possible with an impedance device, and/or (b) known metastatic disease, recurrence of cancer, or lymphedema due to cancer recurrence, or being diagnosed with and treated for lymphedema, or other bulk disease in the thoracic or cervical regions. Of the 356 patients enrolled, 2 were excluded from this data analysis due to incomplete data.

### Variables and Measures

#### Demographic and clinical data

Demographic data included: age, education, marital status, employment status, and ethnicity. Medical records were reviewed to obtain information on: breast cancer diagnosis, stage of the disease, cancer location, type of surgery (mastectomy versus lumpectomy), lymph node procedures (sentinel lymph node biopsy, axillary lymph node dissection or both), type of adjuvant therapy (radiation and chemotherapy), and years since breast cancer diagnosis.

#### Fatigue

The 4-item Vitality Subscale of the 36-Item Short Form Health Survey (SF-36-VS) was used to assess fatigue.^17,18^ The SF-36-VS has good validity and reliability in patients with cancer.^19,20^ The subscale assesses fatigue in terms of how much of the time during the past 4 weeks patients have felt full of life, full of energy, felt worn out, or felt tired. Responses are scored on a 0 to 100 scale, with higher scores indicating less fatigue. Scores of ≤ 50 have been established as a marker for clinically meaningful fatigue.^21^

#### Lymphatic pain

Lymphatic pain was defined as the co-occurrence of pain and swelling in the affected ipsilateral upper limb following breast cancer treatment.^6^ We operationalized lymphatic pain as the self-report of co-occurring pain sensations (ie, pain, aching, soreness) and swelling in the affected ipsilateral upper limb. Lymphatic pain was assessed using *The Breast Cancer and Lymphedema Symptom Experience Index (BCLE-SEI) Part I*.^5,6,9,21,22^ This valid and reliable self-report instrument has a Cronbach’s alpha of 0.92 for symptom occurrence. A response frame of the past three months was used to ensure that the symptoms were persistent. Each item was rated on a 5-point Likert scale (ie, 0 = no presence of a given symptom to 4 = greatest severity of a given symptom). Higher scores indicate more severe lymphatic pain.

#### Activities of daily living (ADLs)

The ADLs subscale from *BCLE-SEI Part II* was used in this study.^21-23^ The ADLs subscale assesses self-reported difficulty in performing thirteen ADLs, (ie, cooking, using a knife, writing, cleaning the house, vacuuming, laundry, bathing, caring for kids, lifting, yard work, dressing, driving, and making the bed). Each item was rated on a 5-point Likert scale (ie, 0 = no difficulty, 1 = a little, 2 = somewhat, 3 = quite a bit, and 4 = a lot). Patients were asked to indicate if a particular activity did not apply to them (eg, if a patient did not have children, the item about caring for kids did not apply). Scores were summed to a possible total of 52 with higher scores indicating higher impairment in ADLs. The 13-item ADLs subscale has a Cronbach’s alpha of 0.94.

#### Emotional distress

The emotional distress subscale from the *BCLE-SEI Part II* was used in this study.^21-22^ This 12-item subscale assesses emotional distress (ie, the negative emotions evoked by an individual’s experience of physical symptoms). Emotional distress encompasses being frustrated, sad, guilt/self-blame, worried, irritable, fear, angry, lonely, helpless, hopeless, anxious, and depressed. Each item was rated on a 5-point Likert scale (ie, 0 = no, 1 = a little, 2 = somewhat, 3 = quite a bit, and 4 = a lot). Scores were summed to a possible total of 48 with higher scores indicating higher levels of emotional distress. Cronbach’s alpha for the emotional distress subscale was 0.91.

#### Overall health

The General Health Subscale of the SF-36 was used to assess overall health.^17,24^ This subscale includes 1 item that asks patients to rate their overall general health from 1 (poor) to 5 (excellent) and 4 additional questions that ask patients to rate whether they get sick easier than other people, are as healthy as other people, are expecting health to worsen, and have excellent health on a scale of 1 (definitely true) to 5 (definitely false). Raw scores were converted to a standardized score that ranged from 0 to 100 based on the instructions in the SF-36 manual. The higher overall health scores indicate better overall health.^24^

#### Anthropometric measurements

Height was measured without shoes to the nearest 0.1 cm using a digital stadiometer (Seca Corporation, Chino, California, USA). A stand-on bioimpedance analysis (BIA) device (InBody 520, Biospace Co., Ltd, Cerritos, CA, USA) was used to measure weight without shoes to the nearest 0.05 kilograms (kg). This device automatically calculated BMI (kilogram/meters squared, kg/m^2^).

### Study Procedures and Data Collection

All measures were completed during a single in-person visit to NYU Langone Perlmutter Cancer Center. All of the self-report questionnaires were administered to the patients using a study iPad connected to the study specific electronic database capture system. To ensure accurate measurement using the BIA device, patients were instructed to stay hydrated; not participate in vigorous weight lifting, aerobic exercise, or hot yoga; not use a sauna; and not consume alcohol for 24 hours prior to their study visit. Patients were instructed to limit exercise to leisure paced walking and not to consume caffeine or food (water was encouraged) within 2 hours prior to their appointment.

### Data Analysis

Data were analyzed using Stata 16 SE (StataCorp LLC, College Station, Texas, US). For this study, 4 symptom groups were created: Group 1: no symptoms (ie, patients had SF-36-VS scores of >50 and reported no lymphatic pain); Group 2: only lymphatic pain (ie, patients reported pain sensations and swelling in the ipsilateral upper limb and SF-36-VS scores of >50); Group 3: only fatigue (ie, patients had SF-36-VS scores of ≤50 but no lymphatic pain); and Group 4: co-occurring fatigue and lymphatic pain (ie, patients had SF-36-VS scores of ≤50 and reported lymphatic pain).

Medians and interquartile ranges (IQR) were calculated for continuous variables and frequencies for categorical variables. Group differences in demographic and clinical characteristics were evaluated using Kruskal–Wallis and Chi-Square tests, with Holm-adjusted Chi-square and Dunn’s post hoc analyses for pairwise comparisons.

To examine the effects of co-occurring fatigue and lymphatic pain on ADLs and emotional distress, 2-part multivariable regression models were used because of the zero inflation of the outcome variables (ie, ADLs and emotional distress).^25^ The first part of the models used a multivariable logistic regression to predict the likelihood of a non-zero (ie, having impaired ADLs or emotional distress) versus a zero (ie, no impaired ADLs or no emotional distress) on the outcome variables of ADLs and emotional distress. The second part of the models used ordinary least squares (OLS) regression to predict the magnitude of the effects of the symptoms within patients who reported a non-zero value (ie, having impaired ADLs or emotional distress). For the outcome of overall health, OLS regression was used because no zero inflation was found.

Potential confounders included in the regression analyses were demographic and clinical characteristics that are associated with fatigue and chronic cancer pain.^5-6,9,10,15-16^ The demographic covariates were: age, BMI, ethnicity, marital status, level of education, and employment status. The clinical covariates included type of cancer surgery (mastectomy versus lumpectomy), type of lymph node procedure (sentinel lymph node biopsy, axillary lymph node dissection, or both), number of lymph nodes removed, receipt of radiation and/or chemotherapy, and years since breast cancer diagnosis. All the tests were conducted at 0.05 alpha level and 95% confidence interval (CI).

## Results

### Demographic and Clinical Characteristics

As shown in Table 1, patients (n = 345) were women who had a median age of 59 years (IQR = 16; range = 26-82). Among the 345 patients, 76% had a bachelor’s or graduate degree, 62% were married or partnered, 65% were employed, and 25% were non-white. In terms of clinical characteristics, 57% of the patients had a lumpectomy, 49% a mastectomy, 61% chemotherapy, and 71% radiotherapy. While 13% of the patients underwent an axillary lymph node dissection, and 45% had a sentinel lymph node biopsy, 43% had both procedures. The median number of lymph nodes removed was 4 (IQR = 10.00; range = 1-35). The median years elapsed since the breast cancer diagnosis was 3 (IQR = 6; range = 0-43 years).

**Table 1.** Demographic and Clinical Characteristics (N = 354).

Characteristics	Total sample (n = 354)		No symptoms (Group 1) (n = 119, 33.6%)		Only Lymphatic Pain (Group 2) (n = 158, 44.6%)		Only Fatigue (Group 3) (n = 20, 5.6%)		Co-occurring Fatigue and Lymphatic Pain (Group 4) (n = 57, 16.1%)		Group comparisons
	Median	*IQR* ^1^	Median	*IQR*	Median	*IQR*	Median	*IQR*	Median	*IQR*	Test Statistics (df) and *P*-values^2^
Age (in years)	59	*16*	62	*14*	58	*15*	61	*25*	54	*15*	*H*(3)=15.6, *P* = .001 4 < 1: *P* < .001 4 < 3: *P* = .042
Body mass index (BMI)	25	*6*	24	*6*	26	*6*	24	*6*	26	*10*	*H*(3)=16.2, *P* = .001 4 > 1: *P* = .002 2 > 1: *P* = .034
Number of lymph nodes removed	4	*10*	4	*8*	4	*10*	6	*10*	6	*11*	*H*(3)=6.27, *P* = .099
Years elapsed since breast cancer diagnosis	3	*6*	3	*7*	3	*6*	3	*4*	2	*2*	*H*(3)=9.31, *P* = .025 4 < 1: *P* = .019
SF-36-VS Fatigue Scores^4^	70	*30*	75	*20*	70	*20*	25	*21*	35	*20*	*H*(3)=185.9, *P* < .001 4 < 1: *P* < .001 4 < 2: *P* < .001 3 < 1: *P* < .001 3 < 2: *P* < .001
Lymphatic pain scores	4	*8*	0	*1*	7	*6*	1	*2*	11	*8*	*H*(3)=215.5, *P* < .001 4 > 1: *P* < .001 4 > 2: *P* = .003 4 > 3: *P* < .001 3 < 2: *P* < .001 2 > 1: *P* < .001
	**n**	*%*	**n**	*%*	**n**	*%*	**n**	*%*	**n**	*%*	**Test Statistics (df) and *P*-values** ^3^
Level of education											χ^2^(15)=24.4, *P* = .058
Less than high school	1	*0*	0	*0*	0	*0*	0	*0*	1	*2*	
High school degree	17	*5*	8	*7*	7	*4*	0	*0*	2	*4*	
Technical school/professional degree	26	*7*	8	*7*	14	*9*	1	*5*	3	*5*	
Associate degree/partial college	40	*11*	7	*6*	21	*13*	0	*0*	12	*21*	
Bachelor’s degree	138	*39*	44	*37*	59	*37*	10	*50*	25	*44*	
Post-bachelor’s degree	132	*37*	52	*44*	57	*36*	9	*45*	14	*25*	
Marital status											χ^2^(9)=14.7, *P* = .098
Married/partnered	221	*62*	70	*59*	106	*67*	14	*70*	31	*54*	
Divorced/separated	49	*14*	19	*16*	18	*11*	3	*15*	9	*16*	
Widowed	22	*6*	8	*7*	13	*8*	1	*5*	0	*0*	
Single/never partnered	62	*18*	22	*18*	21	*13*	2	*10*	17	*30*	
Ethnicity											χ^2^(3)=1.82, *P* = .610
Non-white	88	*25*	29	*24*	37	*23*	4	*20*	18	*32*	
White	266	*75*	90	*76*	121	*77*	16	*80*	39	*68*	
Employment status											χ^2^(3)=2.70, *P* = .440
Unemployed	124	*35*	47	*39*	49	*31*	6	*30*	22	*39*	
Employed	230	*65*	72	*61*	109	*69*	14	*70*	35	*61*	
Radiotherapy											χ^2^(3)=4.08, *P* = .253
Yes	250	*71*	78	*66*	118	*75*	12	*60*	42	*74*	
No	104	*29*	41	*34*	40	*25*	8	*40*	15	*26*	
Chemotherapy											χ^2^(3)=4.50, *P* = .213
Yes	215	*61*	64	*54*	102	*65*	11	*55*	38	*67*	
No	139	*39*	55	*46*	56	*35*	9	*45*	19	*33*	
Mastectomy											χ^2^(3)=3.51, *P* = .319
Yes	173	*49*	57	*48*	72	*46*	13	*65*	31	*54*	
No	181	*51*	62	*52*	86	*54*	7	*35*	26	*46*	
	**n**	*%*	**n**	*%*	**n**	*%*	**n**	*%*	**n**	*%*	**Test Statistics (df) and *P*-values** ^3^
Lumpectomy											χ^2^(3)=0.98, *P* = .806
Yes	201	*57*	69	*58*	92	*58*	10	*50*	30	*53*	
No	153	*43*	50	*42*	66	*42*	10	*50*	27	*47*	
Axillary lymph node dissection											χ^2^(3)=11.4, *P* = .010
Yes	45	*13*	8	*7*	21	*14*	2	*10*	14	*25*	
No	309	*87*	112	*93*	136	*86*	18	*90*	43	*75*	
Sentinel lymph node dissection											χ^2^(3)=5.16, *P* = .160
Yes	160	*45*	58	*48*	75	*47*	9	*45*	18	*32*	
No	194	*55*	62	*52*	83	*53*	11	*55*	39	*68*	
Sentinel lymph node biopsy plus Axillary dissection											χ^2^(3)=1.23, *P* = .745
Yes	149	*42*	54	*45*	61	*39*	9	*45*	25	*44*	
No	205	*58*	66	*55*	96	*61*	11	*55*	32	*56*	

1 IQR = interquartile range.

2 Kruskal–Wallis tests, with Holm-adjusted Dunn’s test for post-hoc comparisons.

3 Pearson’s χ^2^ tests, with Holm-adjusted χ^2^ tests for post-hoc comparisons.

4 Lower values correspond to higher levels of fatigue; scores 50 and below correspond to clinically significant fatigue.

### Co-occurring Fatigue and Lymphatic Pain

Of the 354 patients, 16.1% had co-occurring fatigue and lymphatic pain, 44.6% had only lymphatic pain, 5.6% had only fatigue, and 33.6% had neither fatigue nor lymphatic pain. The only fatigue group (median = 25; IQR = 21.2; range = 10-45) had lowest SF-36-VS scores (ie, worst fatigue), compared to the co-occurring fatigue and lymphatic pain group (median = 35; IQR = 20; range = 0-45), the only lymphatic pain (median = 70; IQR = 20; range = 50-100; *Z* = 7.12, *P* < .001) and the no symptoms (median = 75; IQR = 20; range = 50-100; *Z* = 8.03, *P* < 0.001) groups. No significant difference in SF-36-VS scores were found between the only fatigue group and the co-occurring fatigue and lymphatic pain group (median = 35; IQR = 20; range = 0-45), indicating that patients in both groups experienced comparable severity of fatigue. The no symptoms and only lymphatic pain groups had SF-36-VS scores >50, indicating no fatigue.

The median lymphatic pain score of the total patient sample was 4 (IQR = 8; range = 0-19). Patients in the co-occurring fatigue and lymphatic pain group (median = 11; IQR = 8; range = 1-19) had significantly higher lymphatic pain scores compared to patients in the only lymphatic pain (median = 7; IQR = 6; range = 1-19; *Z* = 3.18, *P* = .003), only fatigue (median = 1; IQR = 2.3; range = 0-11; *Z* = 6.71, *P* < .001) groups.

Patients with co-occurring fatigue and lymphatic pain were younger (median = 54; IQR = 15; range = 30-75) than those in only fatigue (median = 60.5; IQR = 25; range = 34-77; *Z* = 2.64, *P* = .042) and no symptoms (median = 62; IQR = 14; range = 33-82; *Z* = 3.96, *P* < .001) groups. Patients with co-occurring fatigue and lymphatic pain (median = 26.2; IQR = 9.7; range = 18.3-58.6; *Z* = 3.63, *P* = .002) and patients with only lymphatic pain (median = 25.6; IQR = 5.8; range = 17.7-42.9; *Z* = 2.71, *P* = .034) had higher BMI than those in the no symptoms group (median = 23.6; IQR = 6; range = 16-42.6). Patients with co-occurring fatigue and lymphatic pain were closer to their breast cancer diagnosis (median years since diagnosis = 1.8; IQR = 2.2; range = 0-19) than patients with no symptoms (median = 3.3; IQR = 6.7; range = 0-43; *Z* = 2.94, *P* = .019).

### Activities of Daily Living (ADLs)

As shown in Table 2, among the 4 symptom groups, patients in the co-occurring fatigue and lymphatic pain group had higher ADL scores (ie, more impaired ADLs) (median = 11; IQR = 6; range = 0-36) than patients in the only lymphatic pain (median = 3; IQR = 8; range = 0-32; *Z* = 3.73, *P* < .001) and only fatigue (median = 2.5; IQR = 4.5; range = 0-21; *Z* = 3.68, *P* < .001) groups. Table 3 presents the unadjusted and adjusted multivariable regression models. The adjusted multivariable logistic regression model (χ^2^(18) = 71.03, *P* < .001) showed that patients in the co-occurring fatigue and lymphatic pain group had higher odds (OR = 24.43, CI = [5.44-109.67], *P* < .001) of having impaired ADLs than patients in only lymphatic pain group (OR = 4.74, CI = [2.65-8.50], *P* < .001). Patients in the only fatigue group had no significant risk of having impaired ADLs. In terms of magnitude of the effect of the symptoms on ADLs, patients with co-occurring fatigue and lymphatic pain had an overall 179% increase in the ADLs scores (ie, more impaired ADLs; B = 8.06, CI = [5.54-10.59]) compared to patients with no symptoms (*B*_0_ = 4.50, CI = [2.75-6.25]). Both being non-white (B = -2.86, 95% CI −4.77 to −0.95) and having a higher BMI (B = .20, CI = [0.06-0.35]) were associated with more impaired ADLs. The adjusted model explained 22% of the variance in ADL scores (*F* [18, 238] = 3.94, *P* < .001; *R*^2^ = .22).

**Table 2.** Differences in Activities of Daily Living, Emotional Distress, and Overall Health Among Symptom Groups.

Characteristics	Total Sample (n = 354)		No Symptom (1) (n = 119, 33.6%)		Only Lymphatic pain (2) (n = 158, 44.6%)		Only Fatigue (3) (n = 20, 5.6%)		Co-occurring fatigue and lymphatic pain (4) (n = 57, 16.1%)		Group comparisons
	Median	*IQR*	Median	*IQR*	Median	*IQR*	Median	*IQR*	Median	*IQR*	Test statistics (df) and *P*-values^a^
Activities of daily living	3	*8*	1	*3*	3	*8*	3	*5*	11	*16*	*H*(3) = 74.2, *P* < .001 4 > 1: *P* < .001 4 > 2: *P* < .001 4 > 3: *P* < .001 2 > 1: *P* < .001
Emotional distress	1	*5*	0	*0*	2	*6*	0	*4*	8	*14*	*H*(3) = 110.6, *P* < .001 4 > 1: *P* < .001 4 > 2: *P* < .001 4 > 3: *P* < .001 2 > 1: *P* < .001
Overall health	75	*29*	80	*20*	75	*25*	53	*21*	55	*35*	*H*(3) = 73.9, *P* < .001 4 < 1: *P* < .001 4 < 2: *P* < .001 3 < 1: *P* < .001 3 < 2: *P* < .001 2 < 1: *P* = .021

a Kruskal-Wallis tests, with Holm-adjusted Dunn’s test for post hoc comparisons.

**Table 3.** Unadjusted and Adjusted 2-Part Multivariable Regression Model Analysis Predicting the Effect of Co-occurring Fatigue and Lymphatic Pain on Activities of Daily Living (ADLs) (n = 354).

Multivariable logistic regression	Unadjusted			Adjusted^1^		
	OR	95% CI^2^	*P*-value	OR	95% CI	*P*-value
Comparison groups						
No symptom	—	—	—	—	—	—
Only lymphatic pain	4.57	2.65 to 7.87	<.001	4.74	2.65 to 8.50	<.001
Only fatigue	1.48	0.56 to 3.87	.429	1.45	.53 to 4.01	.472
Co-occurring fatigue and lymphatic pain	27.04	6.31 to 115.97	<.001	24.43	5.44 to 109.67	<.001
	Pseudo R^2^	χ^2^ (df)	Prob>χ^2^	Pseudo R^2^	χ^2^ (df)	Prob>χ^2^
	0.14	58.92 (3)	<.001	.17	71.03 (18)	< .001
Ordinary least square regression	B	95% CI	P-value	Coefficient	95% CI	P-value
Comparison groups						
No symptom	—^3^	—	—	—	—	—
Only lymphatic pain	2.55	0.44 to 4.66	.018	1.80	-0.37 to 3.96	.103
Only fatigue	2.25	−2.03 to 6.53	.302	1.65	-2.72 to 6.02	.459
Co-occurring fatigue and lymphatic pain	8.06	5.54 to 10.59	<.001	6.46	3.73 to 9.19	<.001
White				−2.86	−4.77 to −0.95	.003
BMI				.20	0.06 to 0.35	.007
Intercept^3^	4.50	2.75 to 6.25	< .001			
	*R* ^2^	*F* (*df*_1_, *df*_2_)	Prob > F	*R* ^2^	*F* (*df*_1_,*df*_2_)	Prob > F
	.14	13.87 (3, 253)	< .001	.22	3.94 (18, 238)	< .001

1 Adjusted for age, BMI, ethnicity, education, number of lymph nodes removed, having a sentinel lymph biopsy, having an axillary lymph dissection, having a sentinel lymph biopsy plus axillary lymph dissection, having a mastectomy, having a lumpectomy, having radiation, having chemotherapy, and years elapsed since breast cancer treatment. Only statistically significant confounders are shown in the table.

2 OLS: ordinary least square; OR: odds ratio; CI: confidence interval;—: Reference group.

3 Intercept is the average score for women without co-occurring fatigue and pain.

### Emotional Distress

As shown in Table 2, among the 4 symptom groups, patients with co-occurring fatigue and lymphatic pain had higher emotional distress scores (median = 8; IQR = 14; range = 0-48) than those in only fatigue (median = 0; IQR = 4; range = 0-23; *Z* = 4.10, *P* < .001) and only lymphatic pain (median = 2; IQR = 6; range = 0-35; *Z* = 4.52, *P* < .001) groups. Table 4 presents the unadjusted and adjusted multivariable regression models. The adjusted multivariable logistic regression model (χ^2^(18) = 142.85, *P* < .001) demonstrated that patients with co-occurring fatigue and lymphatic pain had the highest odds of having emotional distress (OR = 26.52, CI = [9.64-72.90], *P* < .001), followed by those with only lymphatic pain (OR = 12.82, CI = [6.72-24.46], *P* < .001). Younger age (OR = 0.95, CI = [0.93-0.98], *P* < .001), having axillary lymph node dissection (OR = 0.13, CI = [0.02-0.93, *P* < .043), and having more lymph nodes removed (OR = 1.16 CI = [1.00-1.35], *P* < .046) were associated with higher emotional distress. In terms of magnitude of the effect of the symptoms on emotional distress, patients with co-occurring fatigue and lymphatic pain had an overall 211% increase in emotional distress scores (B = 9.17, CI = [5.52-12.83]) compared to patients with no symptoms (B_0_ = 4.35, CI = [1.38-7.31]). The adjusted model explained 23% of the variance in emotional distress scores (*F* [18, 181] = 3.14, *P* < .001; *R*^2^ = .23).

**Table 4.** Unadjusted and Adjusted 2-part Multivariable Regression Model Analysis Predicting the Effect of Co-Occurring Fatigue and Lymphatic Pain on Emotional Distress (n = 354).

Multivariable logistic regression	Unadjusted			Adjusted^1^		
	OR	95% CI^2^	*P*-value	OR	95% CI	*P*-value
Comparison groups						
No symptom	—	—	—	—	—	—
Only Lymphatic Pain	9.57	5.47 to 16.72	<.001	12.82	6.72-24.46	<.001
Only Fatigue	2.93	1.10 to 7.82	.032	2.44	.84-7.12	.102
Co-occurring fatigue and lymphatic pain	25.55	10.36-63.00	<.001	26.52	9.64-72.90	<.001
Age				.95	.93-.98	<.001
Axillary lymph node dissection				.13	.02-0.93	.043
Lymph nodes removed				1.16	1.00-1.35	.046
	Pseudo R^2^	χ^2^ (df)	Prob>χ^2^	Pseudo R^2^	χ^2^ (df)	Prob>χ^2^
	0.22	104.84 (3)	<.001	.29	142.85 (18)	<.001
Ordinary least square regression	*B*	95% CI	P-value	B	95% CI	*P*-value
Comparison groups						
No symptom	—	—	—	—	—	—
Only Lymphatic Pain	1.21	−2.07 to 4.49	.470	.79	−2.68-4.26	.655
Only Fatigue	4.21	−1.64 to 10.06	.158	3.63	−2.67-9.93	.258
Co-occurring fatigue and lymphatic pain	9.17	5.52 to 12.83	< .001	7.95	4.03-11.86	<.001
Intercept^3^	4.35	1.38-7.31	.004			
	*R* ^2^	*F* (*df*_1_, *df*_2_)	Prob > *F*	*R* ^2^	*F*(*df*_1_, *df*_2_)	Prob > F
	.17	14.09 (3, 196)	<0.001	.23	3.14 (18, 181)	<.001

1 Adjusted for age, BMI, ethnicity, education, number of lymph nodes removed, having a sentinel lymph biopsy, having an axillary lymph dissection, having a sentinel lymph biopsy plus axillary lymph dissection, having a mastectomy, having a lumpectomy, having radiation, having chemotherapy, and years elapsed since breast cancer treatment. Only statistically significant confounders are shown in the table.

2 OLS: ordinary least square; OR: odds ratio; CI: confidence interval;—: Reference group.

3 Intercept is the average score for women without co-occurring fatigue and pain.

### Overall Health

As shown in Table 2, among the 4 symptom groups, patients with co-occurring fatigue and lymphatic pain (median = 55; IQR = 35; range = 10-100) and patients with only fatigue (median = 52.5; IQR = 21.2; range = 0-100) had equivalently lower overall health scores, indicating similarly poorer overall health. Table 5 presents the unadjusted and adjusted OLS models. The adjusted model demonstrated that only fatigue (B = -25.21, CI = [−33.51 to −16.91]), co-occurring pain and fatigue (B = -22.67, CI = [−28.62 to −16.71], and only lymphatic pain (B = −5.78,

**Table 5.** Unadjusted and Adjusted Ordinary Least Square Regression Predicting the Effect of Co-Occurring Fatigue and Lymphatic Pain on Overall Health (n = 354).

	Unadjusted			Adjusted^1^		
	*B*	95% CI^2^	*P*-value	*B*	95% CI	*P*-value
Comparison groups						
No symptom	—	—	—	—	—	—
Only Lymphatic Pain	−6.49	−10.72 to −2.27	<.001	−5.78	−10.06 to −1.51	.008
Only Fatigue	−25.74	−34.14 to −17.33	<.001	−25.21	−33.51 to −16.91	<.001
Co-occurring fatigue and lymphatic pain	−26.29	−31.90 to −20.69	<.001	−22.67	−28.62 to −16.71	<.001
BMI				−0.73	−1.07 to −0.39	<.001
Mastectomy				−10.11	−18.69 to −1.54	.021
Lumpectomy				−11.55	−20.00 to −3.09	.008
Intercept^3^	78.49	75.30 to 81.68	<.001			
	*R* ^2^	*F* (*df*_1_, *df*_2_)	Prob>F	R^2^	*F* (*df*_1_, *df*_2_)	Prob > F
	.23	35.41 (3, 350)	<.001	.30	8.06 (18, 335)	<.001

1 Adjusted for age, BMI, ethnicity, education, number of lymph nodes removed, having a sentinel lymph biopsy, having an axillary lymph dissection, having a sentinel lymph biopsy plus axillary lymph dissection, having a mastectomy, having a lumpectomy, having radiation, having chemotherapy, and years elapsed since breast cancer treatment. Only statistically significant confounders are shown in the table.

2 OLS: ordinary least square; OR: odds ratio; CI: confidence interval;—: Reference group.

3 Intercept is the average score for women without co-occurring fatigue and pain.

CI = [−10.06 to −1.51]) were significant predictors of lower overall health scores. Other significant predictors of overall health included BMI (B = −0.73), having a mastectomy (B = −10.11), and having a lumpectomy (B = −11.55). The adjusted model explained 30% of the variance in overall health scores (*F* [18.335] = 8.06, *P* < .001; *R*^2^ = .30).

## Discussion

This study is the first to provide initial evidence of negative effects of co-occurring fatigue and lymphatic pain on breast cancer patients’ ADLs, emotional distress, and overall health. Our study found that 66.4% of patients experienced either only lymphatic pain (44.6%), co-occurring fatigue and lymphatic pain (16.1%), or only fatigue (5.6%). Consistent with prior studies that observed that approximately 20% of women reported fatigue and 61% reported lymphatic pain,^6,9,11,13,16^ our findings provide additional evidence that women treated for breast cancer continue to experience fatigue and lymphatic pain years after the completion of treatments.

Previous research found that traditional measures of ADLs (eg, toileting, ambulation, continence, and feeding) were less relevant to breast cancer patients in terms of physical function.^23,27^ Our study is the first to demonstrate the incremental negative effects of co-occurring fatigue and lymphatic pain on ADLs among patients without a diagnosis of lymphedema. This finding extends recent work on the negative effects of increased limb volumes on ADLs^21^ as well as prior studies showing that chronic pain and lymphedema are predictors of impaired physical function.^3,15,28^ It should be noted that the experience of only lymphatic pain and co-occurring fatigue and lymphatic pain were significant predictors for impaired ADLs while the experience of only fatigue was not. These findings support our hypothesis that co-occurring fatigue and lymphatic pain have incremental negative effects on impaired ADLs. As a significant risk factor for impairments in ADLs, higher BMI can be modified through lifestyle changes. Because obesity, lymphatic pain, and fatigue are inflammatory conditions,^5,26-28^ future research should explore the interactions among these conditions and their underlying mechanisms. This knowledge will provide directions for interventions for fatigue and lymphatic pain.

Approximately 20% to 40% of breast cancer patients reported negative emotions.^29^ Previous studies found that younger age, more extended disease, more extended surgery, receipt of chemotherapy, poor body image, presence of lymphedema, pain and impaired mobility were risk factors for emotional distress in breast cancer patients.^29-34^ In addition, worst pain severity profiles were associated with significant stress and multiple co-occurring symptoms.^35^ Our study focused on the emotional distress associated with co-occurring symptoms defined as the negative emotions or feelings evoked by an individual’s experience of symptoms.^21,22^ Findings from our study demonstrated the incremental negative effects of co-occurring fatigue and lymphatic pain on emotional distress. It should be noted that the experience of only lymphatic pain and co-occurring fatigue and lymphatic pain were significant predictors of emotional distress while the experience of only fatigue was not. These findings support our hypothesis that co-occurring fatigue and lymphatic pain have incremental negative effects on emotional distress in breast cancer patients.

By providing initial evidence that co-occurring fatigue and lymphatic pain have incremental negative effects on breast cancer patients’ ADLs and emotional distress, our study extends previous research that each symptom has negative effect on physical activity, QOL, and survival.^13-15^ It should be noted that among the 4 symptom groups, the patients with co-occurring fatigue and lymphatic pain had worst lymphatic pain. Interestingly, patients in the only fatigue groups had minimal pain. The incremental effects of co-occurring fatigue and lymphatic pain may be the result of synergistic interactions between the 2 symptoms.^16^ As both fatigue and lymphatic pain are associated with increases in inflammatory responses,^26,27^ future research should investigate physiological interactions between these 2 symptoms. Different from our hypothesis, our study found comparable negative effects of only fatigue and co-occurring fatigue and lymphatic pain on overall health. Both groups had comparably poorer overall health.

### Limitations and Strengths of the Study

The cross-sectional study design prevents an evaluation of changes over time in fatigue and lymphatic pain and symptom group memberships. Nevertheless, this study is the first to provide initial evidence of negative effects of co-occurring fatigue and lymphatic pain on ADLs, emotional distress, and overall health. A strength of the study is the use of a valid and reliable instruments to evaluate symptoms, allowing for precision classification of fatigue and lymphatic pain. The breast cancer specific measures of ADLs is another strength of this study.^6,21^ The primary outcome of this study was to investigate the additive effect of fatigue and lymphatic pain on ADLs and emotional distress in breast cancer survivors and not to identify the tumor and treatment-related predictors of these symptoms. For this reason, we did not extract clinicopathological and detailed cancer treatment data from participant records to include in the analyses. Nonetheless, further consideration of tumor-specific features and treatment modalities could further identify those at risk for developing lymphatic pain, fatigue or both in future studies.

## Conclusion

Findings from this study support previous work that fatigue and lymphatic pain affected many breast cancer patients and impact patients’ QOL, physical functions, and survival.^14,15^ Our findings extended existing knowledge and suggest that fatigue and lymphatic pain exerted negative effects on patients’ ADLs, emotional distress, and overall health. Our findings provide the initial evidence that the synergistic interactions between fatigue and lymphatic pain incrementally aggravate the negative effects on ADLs and emotional distress. In clinical practice and research, fatigue and lymphatic pain as well as their impact are usually assessed as separate phenomena. Findings of the study highlight the need to investigate the mechanisms that underlie the co-occurring fatigue and lymphatic pain to develop interventions that target both symptoms. Findings of our study also illuminate the need to conduct assessment on both fatigue and lymphatic pain as well as their impact as part of routine clinical practice and make appropriate referrals (eg, physical therapy, exercises interventions, emotional counseling) to improve breast cancer patients’ QOL and decrease years lost due to disability.
